# Feasibility of applying computerized adaptive testing to the Clinical Medical Science Comprehensive Examination in Korea: a psychometric study

**DOI:** 10.3352/jeehp.2025.22.29

**Published:** 2025-10-01

**Authors:** Jeongwook Choi, Sung-Soo Jung, Eun Kwang Choi, Kyung Sik Kim, Dong Gi Seo

**Affiliations:** 1 Department of Psychology, College of Social Science, Hallym University, Chuncheon, Korea; 2 Department of Internal Medicine, Chungnam National University College of Medicine, Daejeon, Korea; 3 Department of Internal Medicine, Jeju National University College of Medicine, Jeju, Korea; 4 Department of Surgery, Yonsei University College of Medicine, Seoul, Korea; 5 Medical Education Assessment Corporation, Korea Association of Medical Colleges, Seoul, Korea; The Catholic University of Korea, Korea

**Keywords:** Clinical competence, Computerized adaptive testing, Medical students, Psychometrics, Self-assessment, Republic of Korea

## Abstract

**Purpose:**

This study aimed to investigate the feasibility of transitioning the Clinical Medical Science Comprehensive Examination (CMSCE) to computerized adaptive testing (CAT) in Korea, thereby providing greater opportunities for medical students to accurately compare their clinical competencies with peers nationwide and to monitor their own progress.

**Methods:**

A medical self-assessment using CAT was conducted from March to June 2023, involving 1,541 medical students who volunteered from 40 medical colleges in Korea. An item bank consisting of 1,145 items from previously administered CMSCE examinations (2019–2021) hosted by the Medical Education Assessment Corporation was established. Items were selected through 2-stage filtering, based on classical test theory (discrimination index above 0.15) and item response theory (discrimination parameter estimates above 0.6 and difficulty parameter estimates between –5 and +5). Maximum Fisher information was employed as the item selection method, and maximum likelihood estimation was used for ability estimation.

**Results:**

The CAT was successfully administered without significant issues. The stopping rule was set at a standard error of measurement of 0.25, with a maximum of 50 items for ability estimation. The mean ability score was 0.55, with an average of 28 items administered per student. Students at extreme ability levels reached the maximum of 50 items due to the limited availability of items at appropriate difficulty levels.

**Conclusion:**

The medical self-assessment CAT, the first of its kind in Korea, was successfully implemented nationwide without significant problems. These results indicate strong potential for expanding the use of CAT in medical education assessments.

## Graphical abstract

[Fig f4-jeehp-22-29]

## Introduction

### Background/rationale

Computerized adaptive testing (CAT) is widely used in educational and psychological assessments. CAT automatically constructs an appropriate test for each examinee based on their responses to individual items and the estimated ability derived from these responses. Since CAT provides only the items suitable for measuring the examinee’s ability level, it can more accurately assess ability with fewer items compared to paper-based tests (PBT) or computer-based tests (CBT). Moreover, because CAT naturally reduces the number of items exposed to examinees, it offers advantages in terms of test security compared with traditional assessment methods [1,2]. Due to these benefits, CAT has been adopted in licensure examinations, especially medical licensure. It was first introduced in 1994 in the National Council Licensure Examination for Registered Nurses in the United States. In 2007, CAT was implemented in the certification exams of the National Registry of Emergency Medical Technicians in the United States. It has also been applied to the North American Pharmacist Licensure Examination. Furthermore, CAT is now utilized not only in licensure examinations but also in assessing academic achievement in K–12 education in the United States.

The Clinical Medical Science Comprehensive Examination (CMSCE), hosted by the Medical Education Assessment Corporation, is administered twice annually in Korea. It is open to all students from 40 domestic medical colleges, though it is primarily intended for third- and fourth-year students. Each examination consists of 320 newly developed items and is conducted simultaneously nationwide, either in CBT or PBT format depending on the circumstances of each medical school. The primary goal is to evaluate individual learning competencies and identify areas needing improvement. Administered in August and November, the assessment aims to track changes in learning competency over time. The examination also allows medical students to evaluate their competencies relative to their peers. However, since it is held only twice a year, students have limited opportunities to assess their competency levels. To address this limitation, we introduced a medical self-assessment to enable students to evaluate their progress, compare themselves with peers from other medical schools, and adopted CAT as the assessment delivery method in 2023.

### Objectives

The purpose of this study was to investigate the feasibility of transitioning the CMSCE to CAT in order to facilitate student comparisons with peers nationwide as well as with their own previous performances. Additionally, the study aimed to assess the feasibility of applying CAT within the educational setting of Korean medical colleges.

## Methods

### Ethics statement

This study utilized only anonymized student response data, containing no personally identifiable information. As the study was considered part of an educational self-assessment, it did not fall within the scope of typical human subjects research; therefore, neither institutional review board (IRB) approval nor individual informed consent was required. Student responses to test items were the sole data source; accordingly, neither IRB approval nor informed consent was necessary.

### Study design

This study explored the feasibility of applying CAT to the CMSCE and other medical education contexts. Test items from the CMSCE, which is administered twice yearly in CBT or PBT formats, were delivered to students using CAT. The CAT examination (medical self-assessment CAT) was conducted nationwide for volunteer medical students from March to June 2023.

### Setting

Since CAT is relatively new in Korea, most medical school assessment managers are unfamiliar with it, and few have expertise in psychometrics. This medical self-assessment CAT marked the first time assessment managers at each medical college had operated such a system. Providing materials and CAT delivery software in a non-native language would likely have caused confusion and difficulty; therefore, Korean-language CAT delivery software and manuals were essential for smooth administration. Given the novelty of this assessment, stable operation was emphasized. To meet these requirements, the LIVECAT platform—which supports Korean—was adopted and made accessible through The CAT Korea (www.thecatkorea.com). LIVECAT is a web-based platform that enables students to complete the self-assessment on PCs, laptops, or tablets [3].

The specifications of the medical self-assessment CAT were as follows: the theta range was set from –5 to +5. The starting rule was to randomly assign the first 5 items. The maximum Fisher information method was used for item selection, and maximum likelihood estimation was employed for ability estimation. Two stopping rules were applied: a standard error of estimation of 0.25 and a maximum of 50 items [4].

**Fig. 1.**
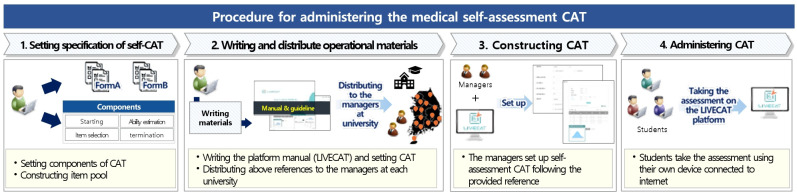
The procedure for administering the medical self-assessment computerized adaptive testing (CAT).

The medical self-assessment CAT procedure involved the following steps (Fig. 1). First, an item pool for the CAT was constructed, and CAT components were specified as described above. Second, a CAT software manual and operational guidelines for assessment managers at each medical college were developed and distributed, providing instructions for configuring CAT components. Third, assessment managers set up the CAT on LIVECAT according to these materials and distributed the assessment uniform resource locator (URL) to students. Finally, students accessed the CAT via the URL, completed the assessment, and received a report of their results.

### Variables

The main variables were the psychometric properties of items (such as difficulty and discrimination indices) and the estimated ability levels of students who participated in the assessment.

### Data source/measurement

Items from the CMSCE administered between 2019 and 2021 were used to establish the item bank for the medical self-assessment CAT. All items were multiple-choice questions with 5 options, and the content covered 7 primary domains: internal medicine, surgery, pediatrics, obstetrics and gynecology, preventive medicine, psychiatry, and clinical specialties.

The medical self-assessment CAT was administered using the LIVECAT platform, with access provided via URLs distributed by medical college managers. The assessments were conducted for 2 weeks each month from March to June 2023, with no time limits imposed during assessment periods. Participation was voluntary, and no examination fee was charged. The response pattern data are presented in Dataset1, and the item estimates for the medical self-assessment CAT are presented in Dataset2.

### Bias

Students who volunteered may differ from non-volunteers in characteristics such as motivation or academic achievement. Therefore, the possibility of self-selection bias must be considered.

### Statistical methods

Items were analyzed using classical test theory (CTT) and item response theory (IRT). Selection was based on both difficulty and discrimination. First, items with a discrimination index above 0.15 in CTT were retained. Next, items with discrimination indices above 0.6 and difficulty parameters between –5 and +5 under IRT were selected. Item discrimination in CTT was calculated using the point-biserial correlation. Items were calibrated using a 2-parameter logistic model. The irtQ and CTT packages in R (The R Foundation for Statistical Computing) were used for item analysis [5,6]. R code for the analyses is provided in Supplement 1.

## Results

### Item analysis

**Fig. 2.**
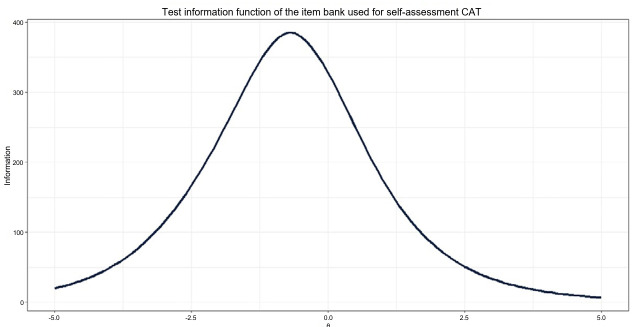
Test information function of the item bank used for self-assessment computerized adaptive testing (CAT).

Based on the selection criteria, 1,145 items were included in the item bank for the medical self-assessment CAT. The number of items in each domain is presented in Table 1. The average item difficulty was –0.70, ranging from –4.21 to 4.10. The average item discrimination was 1.26, ranging from 0.60 to 3.97. Fig. 2 shows the test information function (TIF) of the item bank used in the self-assessment CAT. The TIF peaked at an ability score of –0.7 and declined toward both lower and higher ability levels.

### Perspectives on assessment and scores

**Fig. 3.**
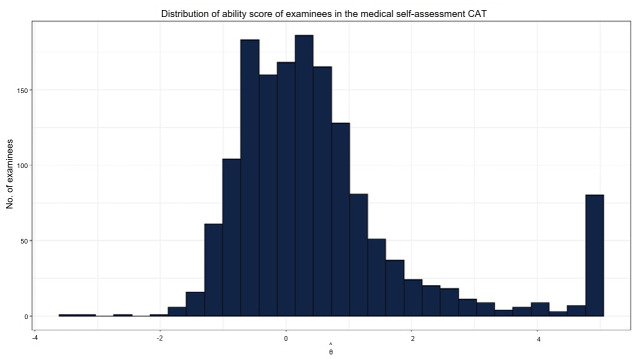
Distribution of ability score of examinees in the medical self-assessment computerized adaptive testing (CAT).

During the medical self-assessment period, a total of 1,541 students participated. The average ability score across all participants was 0.55, with a standard deviation of 1.47. A small number of students answered all items correctly. Overall, the distribution of ability scores closely approximated a normal distribution (Fig. 3). The mean and standard deviation of ability scores across assessment sessions showed minimal variation and remained relatively stable. On average, students completed 28 items. However, those at the lower and higher extremes of ability were administered up to 50 items, the maximum set by the termination rule.

### Perspectives on administering the assessment

Despite being the first implementation of CAT in this setting, the medical self-assessment CAT was carried out smoothly and without major issues. Assessment managers at each medical college, though not psychometricians and initially inexperienced with CAT, successfully constructed and managed the assessments using the provided platform manual and guidelines. Likewise, students—despite their lack of prior exposure to this format—completed the examination without significant concerns or objections.

## Discussion

### Key results

An item bank of 1,145 questions with sound psychometric properties (average difficulty –0.70, discrimination parameter estimate 1.26) was established. A total of 1,541 students participated, and their ability scores followed a normal distribution (mean=0.55, standard deviation=1.47). The CAT was administered smoothly and successfully, even for first-time users, without significant issues.

### Interpretation

The medical self-assessment CAT represents the first official application of CAT in Korea. Despite being the initial implementation, the assessment was conducted simultaneously across all 40 medical colleges without notable difficulties, which is a noteworthy achievement. Several key implications emerge from this experience. First, CAT was applied for the first time in Korea to assess healthcare students, comparable to its use in licensure examinations for pharmacists and paramedics in the United States. Second, students exhibited minimal resistance to the CAT format and viewed it positively as a tool for personalized learning. Finally, these real-world results indicate that CAT holds considerable potential for broader application and implementation in Korean medical education.

### Limitation

For students at the lower or higher ends of the ability spectrum, the maximum of 50 items was reached because of an insufficient number of appropriately difficult items. To rectify this, further development of items targeting both extremes of the ability continuum will be necessary. In particular, more high-difficulty items should be created to adequately measure high-ability students. As shown in Fig. 3, relatively few students were in the low-ability group, whereas more students were observed at higher ability levels. Accordingly, additional items that capture both low- and high-end abilities should be prioritized.

Additionally, the medical self-assessment CAT was conducted without content balancing. Because the content was divided into multiple detailed domains, there were not enough items to implement effective content-balancing constraints. Future efforts should focus on developing a sufficient number of items across all content domains to ensure robust content balancing [2,7].

### Generalizability

Although rooted in the specific context of the Korean Clinical Medical Science Comprehensive Examination (CMSCE), the findings of this feasibility study have considerable generalizability. They may apply to 3 broader areas: the Korean health professions education system as a whole, international educational contexts facing similar challenges, and the operational design of higher-stakes assessments.

### Suggestion for further studies

While the current item bank is functional, it has clear areas for improvement. Future research should systematically expand the bank, particularly at its extremes, through dedicated item development and evaluation projects.

## Conclusion

This study empirically demonstrates the feasibility and scalability of CAT in Korean medical education. The successful deployment of a self-assessment CAT across all 40 medical colleges established a robust item bank and confirmed that non-expert staff could administer the system effectively. This pivotal implementation shifts CAT from a theoretical concept to a demonstrated reality. It provides proof of concept for broader adoption, laying the foundation for more efficient, secure, and personalized assessments.

## Figures and Tables

**Figure f4-jeehp-22-29:**
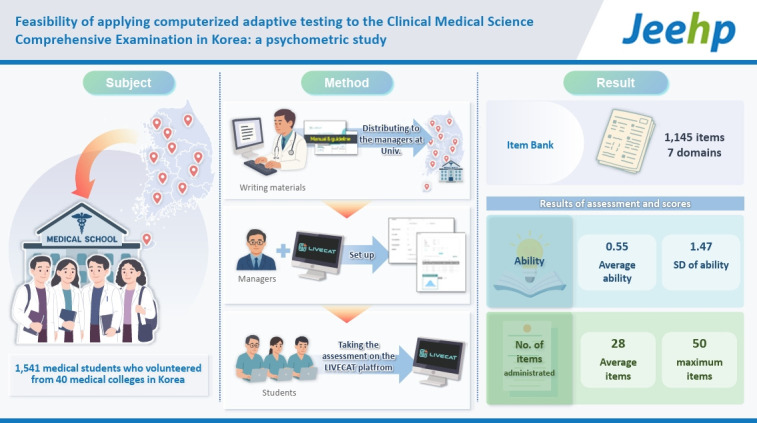


**Table 1.** The number of items in each domain

Domain	No. of items
Internal medicine	515
Surgery	122
Pediatrics	143
Obstetrics and gynecology	134
Preventive medicine	48
Psychiatry	96
Clinical specialty	87
Total	1,145

## Footnotes

**Data availability**
Data files are available from https://doi.org/10.7910/DVN/TQZQ6L Dataset 1. Raw data for responses to items.

Dataset 2. Estimated item parameters for the item bank of medical self-assessment CAT.

## Supplementary materials

Supplement 1. R code for data analysis, which is also available at Github (https://github.com/cat-wook/JEEHP_R_Code.git).

Supplement 2. Audio recording of the abstract.
